# Thyroid cytology Bethesda category III (AUS/FLUS): surgical follow-up and histologic outcomes in a tertiary-care setting

**DOI:** 10.3389/fmed.2026.1799405

**Published:** 2026-03-19

**Authors:** Zgjim Limani, Rinë Limani, Shkelzen Reçica, Labinota Kondirolli, Etnik Bajraktari, Brikenë Blakaj Gashi, Drita Miftari Pazhari

**Affiliations:** 1 Department of Otorhinolaryngology, Faculty of Medicine, University of Prishtina, Prishtina, Kosovo; 2 Department of Anatomical Pathology, Faculty of Medicine, University of Prishtina, Prishtina, Kosovo; 3 Faculty of Medicine, University of Prishtina, Prishtina, Kosovo

**Keywords:** AUS/FLUS, Bethesda, fine-needle aspiration, histology, thyroid, surgical follow-up

## Abstract

**Background and Aim:**

Indeterminate thyroid cytology, specifically category III atypia/follicular lesion of undetermined significance (AUS/FLUS), poses a diagnostic challenge and affects patient management.

**Methods:**

A retrospective review of cases reported as AUS/FLUS was conducted using the institutional thyroid fine needle aspiration (FNA) registry (2013–2022). Proportions were reported with 95% confidence intervals (CIs). Direct registry correlation and a larger all-operated sensitivity denominator were examined to capture real-world outcomes.

**Results:**

AUS/FLUS comprised 243 out of 1,872 FNAs (13.0%; 95% CI: 11.5–14.6). Of these, 186 patients (76.5%; 95% CI: 70.8–81.4) underwent surgery. Histologic follow-up identified 57 malignancies, resulting in a malignancy rate of 41.9% (95% CI: 34.0–50.3) among definitively linked cytology–histology cases (*n* = 136), and a more conservative estimate of 30.6% (95% CI: 24.5–37.6) when considering all operated cases (*n* = 186).

**Conclusion:**

In a tertiary-care setting without routine molecular testing, AUS/FLUS was prevalent and often led to surgical intervention. The malignancy reported rate was clinically significant but varied according to the denominator applied, highlighting the impact of verification and linkage effects and the necessity for clear counseling and ultrasound-informed follow-up strategies.

## 1 Introduction

Fine-needle aspiration (FNA) and ultrasound are the main methods for preoperative assessment of thyroid nodules, informing decisions from monitoring to surgery. The Bethesda System for Reporting Thyroid Cytopathology (TBSRTC) standardizes terminology and links each category to a risk of malignancy and recommended management (1, 2).

Category III atypia/follicular lesion of undetermined significance (AUS/FLUS) is the most varied category and shows the greatest differences between institutions in both its use and its malignancy rates (1–4).

AUS/FLUS includes samples with atypia that are more than benign changes but not enough for a diagnosis of follicular neoplasm, suspicious for malignancy, or malignancy. This category typically encompasses borderline nuclear atypia, limited architectural details, sampling limitations, or atypia occurring in confounding backgrounds such as cystic degeneration, hemorrhage, or lymphocytic thyroiditis (1, 2). The cytologic features in AUS/FLUS are biologically diverse, so this category serves as a broad “risk container” rather than a clear diagnostic group. Minor interpretative variation, specimen quality, and slide preparation may influence category assignment and may consequently influence estimated malignancy risk and clinical management (3–5).

In centers with access to molecular classifiers, targeted mutation panels, or gene expression-based tests can help refine risk assessment in indeterminate cytology and may reduce the need for surgery in some low-risk nodules (6, 7).

However, in many health systems with limited access to molecular testing, risk management relies on repeat FNA, ultrasound-based risk assessment (such as TI-RADS), and selective diagnostic lobectomy based on clinical risk and patient preference (8–10).

In this context, a local estimate of AUS/FLUS outcomes is clinically valuable, not only as a malignancy probability but also as a measure of diagnostic uncertainty and pathway escalation (how often AUS/FLUS triggers repeat testing or surgery). Importantly, observed malignancy rates are sensitive to verification bias such as when surgery is preferentially performed in higher-risk nodules, the operated subgroup becomes enriched for malignancy, thereby increasing the apparent risk (3, 4).

This analysis of AUS/FLUS builds upon previous registry reports by incorporating confidence intervals and providing clear outcome estimates relevant to local decision-making.

Aim: To quantify the frequency of AUS/FLUS, the rate of surgical follow-up, and the rate of malignant histologic outcomes in a tertiary-care setting, and to use these metrics as practical indicators of diagnostic uncertainty.

## 2 Materials and methods

### 2.1 Study design and setting

A retrospective analysis was conducted on category III AUS/FLUS cases documented in a 10-year institutional thyroid FNA registry at the University Clinical Center of Kosovo (UCCK), spanning January 2013 to December 2022. Data were sourced from the previously reported UCCK thyroid FNA registry (11); this manuscript presents an AUS/FLUS-focused analysis with expanded outcome reporting, including Wilson 95% confidence intervals, explicit denominator and linkage transparency, and number needed to operate (NNO) based clinical efficiency metrics.

UCCK is the main tertiary referral center in the national healthcare system. The registry records cytology category at reporting and subsequent clinical disposition, including surgery, allowing evaluation of real-world outcomes in a tertiary-care pathway. To focus on diagnostic uncertainty within Bethesda III, this study was restricted to AUS/FLUS.

### 2.2 FNA procedure and cytology reporting

Thyroid FNAs were performed under ultrasound guidance in routine practice by trained operators, such as endocrinologists or radiologists, depending on local workflow. Aspirates were prepared using standard laboratory protocols and stained with May-Grünwald-Giemsa and/or Papanicolaou.

The adequacy assessment was based on the final slide review.

Cytology reports were issued using TBSRTC. In cases without an explicit Bethesda designation, retrospective reclassification was done based on documented cytomorphologic features for uniform categorization. AUS/FLUS was assigned when atypia exceeded benign changes but did not meet criteria for follicular neoplasm, suspicious for malignancy, or malignancy. Typical triggers for AUS/FLUS include focal nuclear atypia insufficient for Bethesda V/VI, limited architectural information in follicular-patterned aspirates, and atypia in a confounding background such as cystic degeneration or thyroiditis.

### 2.3 Case definition and unit of analysis

AUS/FLUS cases were defined as thyroid FNA reports categorized as Bethesda category III in the registry. Cases were identified by searching the issued reports database for Bethesda III designations. Reports lacking an explicit Bethesda label were also included if the descriptive cytology text met AUS/FLUS criteria and the final impression corresponded to Bethesda category III. In such instances, two pathologists independetly reviewed the report text, and discrepancies were resolved by consensus to minimize misclassification. When multiple FNAs were present for a patient or nodule, the AUS/FLUS episode in the registry was treated as the index event for pathway-level analysis. This methodology was selected to reflect real-world counseling following a Bethesda III result.

#### 2.3.1 Outcomes and cytology–histology correlation

The primary endpoint was final surgical histopathology, classified as benign vs. malignant, among AUS/FLUS cases proceeding to surgery.

##### 2.3.1.1 Histopathology definitions

Malignancy was defined as thyroid carcinoma on final surgical pathology (e.g., papillary thyroid carcinoma, including variants, follicular thyroid carcinoma, medullary thyroid carcinoma, poorly differentiated carcinoma, and anaplastic carcinoma). Non-invasive follicular thyroid neoplasm with papillary-like nuclear features (NIFTP), when reported, was classified as non-malignant neoplasm in the primary analysis and described separately where possible, because its biological behavior and management differ from invasive carcinoma. Incidental papillary thyroid microcarcinomas identified on surgical histology were classified as malignant outcomes (regardless of whether they were the dominant nodule), consistent with routine histopathologic outcome reporting.

Malignancy outcome rate was reported using the denominator applied in the registry correlation tables, reflecting definitive cytology–histology correlation under the registry’s linkage rules (e.g., nodule-level matching and availability of definitive histology in the linked case). Operationally, cases were treated as definitively linked when a unique patient identifier and a nodule-level match (including laterality/clinical context) allowed one-to-one assignment of the index AUS/FLUS FNA to a finalized surgical histopathology record within the institutional system. Surgically treated AUS/FLUS cases were classified as ‘not contributing’ to the correlation denominator when this one-to-one mapping could not be established, most commonly due to incomplete identifiers in the cytology or surgical record, unavailable final histology in the institutional archive (e.g., surgery performed elsewhere), or multinodular disease with uncertain nodule-to-specimen correspondence. Because registry linkage can be incomplete, an all-operated sensitivity estimate was also reported using all AUS/FLUS cases that underwent surgery as the denominator. Secondary pathway endpoints included the proportion of AUS/FLUS cases proceeding to surgery (pathway escalation) and derived efficiency measures (e.g., number needed to operate to identify one malignancy).

### 2.4 Statistical analysis

Data are summarized as counts and proportions. Wilson 95% confidence intervals (CI) were calculated for AUS/FLUS frequency, surgery uptake, and the proportion of malignant histologic outcomes.

To enhance interpretability, denominator transparency was maintained by reporting the proportion of malignant outcomes using both the registry correlation denominator and a conservative all-operated sensitivity estimate. Pathway-level uncertainty was quantified using derived metrics, such as the percentage of all FNAs resulting in AUS/FLUS-related surgery and the number needed to operate.

No hypothesis testing was prespecified; analyses were descriptive and intended to inform counseling and local pathway optimization.

#### 2.4.1 Ethics

This study received approval from the Ethics Committees of the Faculty of Medicine University of Prishtina and the Chamber of Kosovo Medical Doctors (Ref no: 4547, dated 31.05.2023; and Ref no: 101/2023, dated 20.06.2023; respectively). Patient confidentiality was maintained throughout, and data were anonymized prior to analysis. As a retrospective review of previously collected specimens and clinical data, informed consent was waived.

## 3 Results

### 3.1 AUS/FLUS frequency and pathway escalation

**Figure 1:**
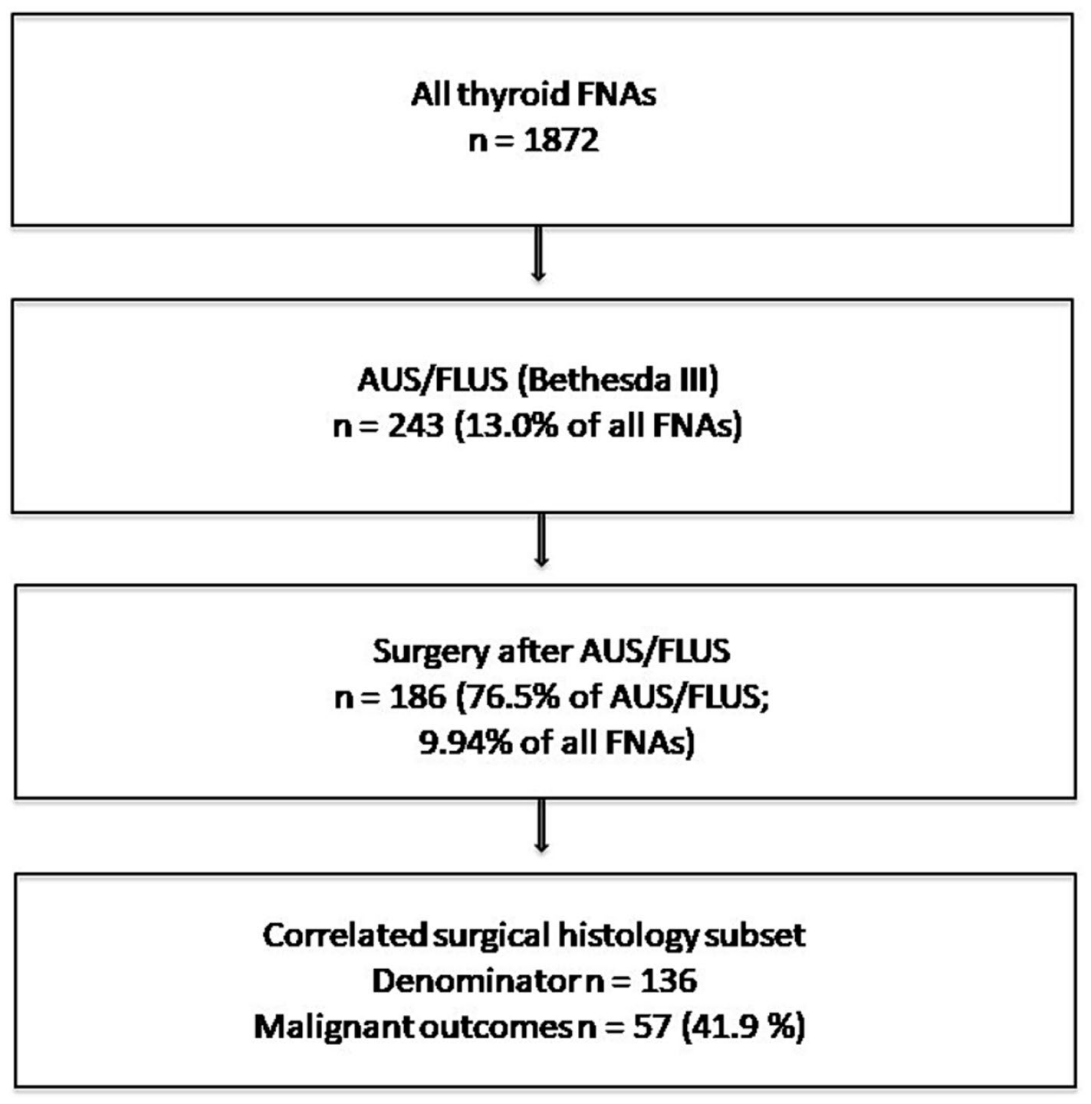
AUS/FLUS pathway flow diagram. The correlation denominator (*n* = 136) reflects registry linkage rules for definitive cytology–histology correlation.

Among 1,872 thyroid FNAs, AUS/FLUS accounted for 243 cases (13.0%; 95% CI: 11.5–14.6). Surgery followed in 186/243 cases (76.5%; 95% CI: 70.8–81.4), indicating that a Bethesda III result led to operative escalation in 186/1,872 FNAs (9.94%; 95% CI: 8.66–11.37). Figure 1 summarizes the AUS/FLUS pathway and denominators used for outcome reporting.

Table 1 summarizes the baseline characteristics of AUS/FLUS (Bethesda III) cases. The cohort was mostly female, with a mean age of 50 years, reflecting the typical demographic for thyroid nodular disease. Most nodules were moderately sized (mean maximum diameter 2.4 cm) and showed no significant laterality. Surgery was common after AUS/FLUS diagnosis, with over three-quarters of cases undergoing resection. About one-quarter of patients who underwent surgery had a repeat FNA before surgery. The median interval from cytologic diagnosis to surgery was short, showing prompt clinical decision-making in this tertiary-care setting.

**Table 1:** Baseline characteristics of AUS/FLUS (Bethesda III) cases.

Variable	AUS/FLUS cohort
AUS/FLUS cases (*n*)	243
Surgery after AUS/FLUS, *n* (%)	186 (76.5%)
Age, years	49.8 ± 13.6 (range 18–82)
Female sex, *n* (%)	181/243 (74.5%)
Nodule size (max diameter), cm	2.4 ± 1.1 (range 0.7–6.2)
Laterality, *n* (%)	Right 116 (47.7%); Left 102 (42.0%); Isthmus 13 (5.3%); Not recorded 12 (5.0%)
Repeat FNA before surgery (among operated), *n* (%)	52/186 (28.0%)
Time from AUS/FLUS to surgery, months	median 3.2 (IQR 1.6–6.5)

Among AUS/FLUS cases with definitive cytology–histology correlation (*n* = 136), 57 (41.9%) were malignant and 79 (58.1%) were non-malignant (including NIFTP). Papillary thyroid carcinoma (classical and follicular variants) accounted for the majority of malignancies (44/57, 77.2%), followed by follicular thyroid carcinoma (9/57, 15.8%); poorly differentiated thyroid carcinoma (1/57, 1.8%) and medullary thyroid carcinoma (3/57, 5.3%) were infrequent. Within the non-malignant group, nodular hyperplasia/colloid nodule/multinodular goiter was most common (38/79, 48.1%), followed by follicular adenoma (27/79, 34.2%), thyroiditis/other benign lesions (11/79, 13.9%), and NIFTP (3/79, 3.8%) (Table 2).

**Table 2:** Histologic outcomes among AUS/FLUS cases with definitive cytology–histology correlation (*n* = 136).

Outcome group	Histological diagnosis	*n*	% within subgroup
Malignant (*n* = 57)	Papillary thyroid carcinoma (classical + follicular variant)	44	77.2%
	Follicular thyroid carcinoma	9	15.8%
	Poorly differentiated thyroid carcinoma	1	1.8%
	Medullary thyroid carcinoma	3	5.3%
	Subtotal (malignant)	57	100%
Non-malignant (incl. NIFTP) (*n* = 79)	Nodular hyperplasia / colloid nodule / multinodular goiter	38	48.1%
	Follicular adenoma	27	34.2%
	NIFTP	3	3.8%
	Thyroiditis / other benign lesions	11	13.9%
	Subtotal (non-malignant)	79	100%

NIFTP was classified as non-malignant for malignancy-rate calculations.

### 3.2 Malignant outcomes and malignancy outcome rate under different denominators

Using the registry cytology–histology correlation dataset (*n* = 136 AUS/FLUS cases with definitive linkage), 57 malignancies were identified (41.9%; 95% CI: 34.0–50.3). When all surgically treated AUS/FLUS cases were considered (*n* = 186), the proportion of malignant outcomes 30.6% (57/186; 95% CI: 24.5–37.6), reflecting inclusion of cases without definitive registry linkage. The minimum observed malignant fraction among all AUS/FLUS diagnoses (without assumptions for non-operated cases) was 23.5% (57/243; 95% CI: 18.6–29.2).

Table 3 summarizes linkage-completeness metrics that contextualize the differences between the correlation-based and all-operated malignancy proportions.

**Table 3:** Diagnostic resolution and linkage completeness within AUS/FLUS.

Metric	*n*/*N*	% (Wilson 95% CI)	Interpretation
Observed malignancies among all AUS/FLUS (minimum malignancy fraction)	57/243	23.5% (18.6–29.2)	Lower-bound malignancy fraction without assumptions for non-operated cases
AUS/FLUS with correlated histology (overall)	136/243	56.0% (49.7–62.1)	Defines denominator for correlation-based yield
Correlation completeness among operated AUS/FLUS	136/186	73.1% (66.3–79.0)	Explains correlation vs. all-operated difference
Operated but not contributing to correlation denominator	50/186	26.9% (21.0–33.7)	Transparency for linkage rules

#### 3.2.1 Clinical efficiency metrics

To express the practical “cost” of diagnostic escalation after AUS/FLUS, the NNO to identify one malignancy was calculated. The NNO was 2.39 under the correlation denominator (136/57) and 3.26 under the all-operated denominator (186/57). Expressed differently, this corresponds to 1.39 benign operations per malignancy identified using correlated cases and 2.26 benign operations per malignancy using the all-operated denominator. AUS/FLUS-associated malignancies represented 3.04% of all FNAs (57/1,872; 95% CI: 2.36–3.92) (Table 4).

**Table 4:** Clinical efficiency metrics derived from AUS/FLUS outcomes.

Metric	Value	Interpretation
NNO — correlation denominator	2.39 (136/57)	Operations required to identify one malignancy among correlated cases
NNO — all operated denominator	3.26 (186/57)	Operations required to identify one malignancy among all operated AUS/FLUS
Benign operations per malignancy — correlation denominator	1.39	Benign surgery burden per malignancy identified using correlated cases
Benign operations per malignancy — all operated denominator	2.26	Benign surgery burden per malignancy identified using all operated cases
AUS/FLUS malignancies among all FNAs	3.04% (57/1,872; 95% CI: 2.36–3.92)	Overall malignant burden attributable to AUS/FLUS within the full FNA cohort

NNO, number needed to operate denominator/number of malignancies. “Benign operations per malignancy” reflects the benign surgery burden per malignancy under the stated denominator.

## 4 Discussion

This AUS/FLUS focused analysis demonstrates that Bethesda category III constitutes a genuine diagnostic grey zone in routine tertiary-care clinical practice. In this context, AUS/FLUS accounted for 13% of all FNAs, with over three-quarters of cases progressing to surgery. The malignancy proportion was clinically significant when reported with confidence intervals and clearly defined denominators.

The relatively high surgical rate following AUS/FLUS (76.5%) likely reflects tertiary-care referral patterns, institutional decision thresholds, and limited molecular refinement rather than intrinsic Bethesda III biology alone. In this setting, nodules selected for surgery may be enriched for higher clinical or ultrasound suspicion, contributing to operative selection and verification effects. These results should therefore be interpreted within the local diagnostic pathway structure. More broadly, the risk attributed to Bethesda III is not a fixed biological property but a composite construct shaped by cytologic thresholds and category heterogeneity. AUS/FLUS encompasses borderline nuclear changes, limited architectural information, and atypia arising in confounding backgrounds such as cystic change or inflammation. Variation in the distribution of these sub-patterns across institutions is expected to produce different malignant outcome rates.

### 4.1 AUS/FLUS heterogeneity and reproducibility

Interobserver variability in assigning AUS/FLUS is a major contributor of institutional differences in the frequency of Bethesda III and the observed proportion of malignant histologic outcomes. Even within a single institution, borderline cases cluster near interpretive thresholds, and differences in specimen preparation, cellularity, and adequacy can further increase variability. Meta-analytic evaluations of Bethesda performance confirm that malignancy risk in AUS/FLUS can diverge from early estimates, particularly in referral-enriched or surgery-selected cohorts, illustrating the importance of local outcome data for counseling and pathway design (3, 4, 12, 13). In addition, evolving histologic terminology, most notably the introduction of NIFTP, can shift apparent malignancy rates over time and complicate cross-study analyses (14).

From a practical pathology perspective, these findings support two complementary strategies, reinforcing internal criteria and peer review for AUS/FLUS assignment to minimize avoidable variability, and integrating ultrasound risk features and clinical context into post-cytology decision making to prevent unnecessary escalation in low-risk imaging and clinically stable nodules.

### 4.2 Implications in settings without routine molecular testing

In this tertiary-care setting, a substantial proportion of AUS/FLUS diagnoses proceeded to surgery, reflecting a pathway that prioritizes diagnostic clarification when clinical or sonographic concern is present over prolonged surveillance. Due to the retrospective registry design, the precise decision sequence leading to surgery (including whether repeat FNA was omitted in individual cases) could not be uniformly reconstructed. However, institutional management practices incorporate ultrasound risk features, clinical context, and feasibility of surveillance alongside cytology results when determining the next step. In the absence of routine molecular risk stratification, clinical decisions relied on integrated assessment of cytology, ultrasound findings, and overall risk context rather than molecular refinement. Consequently, observed malignant outcome rates should be interpreted within the context of local follow-up structure and referral patterns. Transparent reporting of denominators is therefore essential when interpreting real world malignancy estimates.

#### 4.2.1 Pragmatic follow-up pathway

In settings without routine molecular testing, a practical approach after AUS/FLUS is to confirm that ultrasound risk features and clinical context have been carefully reviewed. For nodules without high-risk ultrasound features and for which reliable follow-up is feasible, repeat FNA and short-interval ultrasound reassessment may be prioritized. For nodules with high-risk ultrasound features, significant clinical concern, or unreliable follow-up, early diagnostic lobectomy can be considered. This pathway acknowledges that decisions regarding the next management step are influenced not only by the cytologic category, but also by feasibility of surveillance and patient preference.

### 4.3 Clearly defined denominators and verification bias

A frequent weakness in AUS/FLUS outcome reporting is ambiguity about the denominator (e.g., all operated cases versus only cases with definitive cytology–histology linkage) and limited discussion of verification bias. When surgery is performed preferentially on nodules perceived as higher risk, the operated subgroup becomes enriched for malignancy, leading to an apparent increase in malignancy rate.

For this reason, malignancy outcomes rate was reported using three complementary frames, the correlation denominator (registry-linked definitive histology), a conservative all-operated denominator, and the minimum observed malignant fraction among all AUS/FLUS diagnoses. Presenting these estimates together enables clinicians to interpret risk in context and improves comparability across studies with different linkage rules and follow-up patterns.

Incidental papillary thyroid microcarcinomas identified on final histology were classified as malignant outcomes in accordance with routine histopathologic reporting standards. While this approach reflects real-world surgical pathology practice, inclusion of incidental microcarcinomas may modestly increase the calculated malignancy proportion in surgery-selected cohorts. This consideration is important when comparing reported malignancy rates across AUS/FLUS series, particularly when studies differ in whether incidental microcarcinomas are included or excluded.

### 4.4 Clinical interpretation and counseling

Clinically, AUS/FLUS should be conveyed as a result that signifies genuine diagnostic uncertainty rather than a definitive diagnosis. The appropriate counseling message is that atypical cells are present, but the degree of atypia does not meet criteria for suspicion or malignancy. Consequentely, subsequent management should be guided by ultrasound risk features, clinical symptoms, and the feasibility of repeat testing and follow-up. The reported pathway-level metrics, including progression to surgery and NNO, can facilitate consistent shared decision-making and help align patient expectations with local practice realities.

## 5 Limitations

This single-center retrospective analysis reflects referral and surgical selection patterns typical of a tertiary-care setting, which may not apply to primary or community pathways. Inconsistent availability of ultrasound risk features and detailed repeat FNA category shifts (including persistence of AUS/FLUS or possible upgrades to Bethesda V/VI or downgrade patterns), and absence of routine molecular testing limited stratified analyses and prevented formal evaluation of stepwise risk refinement and cytologic evolution within AUS/FLUS prior to surgery. Registry linkage rules also resulted in a smaller cytology–histology correlation denominator compared to the total number of operated cases. Therefore, malignant outcome rates were reported using clearly defined alternative denominators, including a conservative all-operated estimate.

## 6 Conclusion

Bethesda category III AUS/FLUS constituted a substantial proportion of thyroid FNAs in this institution and was frequently followed by surgical intervention. The proportion of malignant outcomes was clinically meaningful and varied according to the denominator applied, demonstrating how follow-up patterns and linkage completeness shape real world risk estimation. These findings support structured, patient centered counseling and locally feasible follow-up pathways that integrate cytology with ultrasound risk stratification and clinical context.

## Data Availability

The raw data supporting the conclusions of this article will be made available by the authors, without undue reservation.
